# A single aromatic residue in transcriptional repressor protein KorA is critical for cooperativity with its co-regulator KorB

**DOI:** 10.1111/j.1365-2958.2008.06498.x

**Published:** 2008-10-29

**Authors:** Lewis E H Bingle, Karthik V Rajasekar, Sidra tul Muntaha, Vinod Nadella, Eva I Hyde, Christopher M Thomas

**Affiliations:** School of Biosciences, University of Birmingham, EdgbastonBirmingham B15 2TT, UK

## Abstract

A central feature of broad host range IncP-1 plasmids is the set of regulatory circuits that tightly control plasmid core functions under steady-state conditions. Cooperativity between KorB and either KorA or TrbA repressor proteins is a key element of these circuits and deletion analysis has implicated the conserved C-terminal domain of KorA and TrbA in this interaction. By NMR we show that KorA and KorB interact directly and identify KorA amino acids that are affected on KorB binding. Studies on mutants showed that tyrosine 84 (or phenylalanine, in some alleles) is dispensable for repressor activity but critical for the specific interaction with KorB in both *in vivo* reporter gene assays and *in vitro* electrophoretic mobility shift and co-purification assays. This confirms that direct and specific protein–protein interactions are responsible for the cooperativity observed between KorB and its corepressors and lays the basis for determining the biological importance of this cooperativity.

## Introduction

Cooperativity is said to occur when the interaction of two components of a system (for example, binding of a protein to DNA) makes a third activity (for example, the binding of a second protein molecule to the DNA) more or less likely to happen and is a key emergent property in complex systems (Whitty, 2008). Heterologous cooperativity between two or more different DNA binding proteins can allow sensitive channelling of multiple signals into a single gene expression control point. Cooperative interaction is an important theme in eukaryotic transcriptional regulation, with examples known both in activation (Merika and Thanos, 2001) and repression (Valentine *et al*., 1998). Studies of prokaryotic DNA-binding proteins have uncovered only a few examples of heterologous cooperativity (Barnard *et al*., 2004). The best studied interactions of this type in bacteria are probably those between the global ‘catabolite repressor’ CRP and various more local pathway regulators, such as MelR (Wade *et al*., 2001) and CytR (Pedersen *et al*., 1991; Shin *et al*., 2001; Chahla *et al*., 2003) in *Escherichia coli*, or PutR in *Vibrio vulnificus* (Lee and Choi, 2006). These lead to activation or repression of a promoter by direct protein–protein interaction or modulation of local DNA conformation. A classic example of cooperative binding of DNA by a single regulatory protein (homologous cooperativity) involves the cI protein of bacteriophage λ (Dodd *et al*., 2005), where predominantly electrostatic interactions are required for cooperative binding of DNA by two adjacent dimers (Bell *et al*., 2000). In a recent study of the heterologous cooperative interaction between λ cI protein and RNAP (RNA polymerase) that activates transcription, no conformational changes in the activator or the target molecules were observed and electrostatic and hydrogen bond interaction within a small protein–protein interface of up to 6 amino acids (2 from cI and 4 from the sigma subunit of RNAP) was responsible for cooperativity (Jain *et al*., 2004). Direct interaction is also responsible for recruitment of RNAP to the promoter by CRP (Chen *et al*., 1994; Niu *et al*., 1996). Otherwise, detailed information about the protein–protein interactions that lead to heterologous cooperativity in DNA binding by transcriptional regulators is largely absent from the literature.

**Fig. 1:**
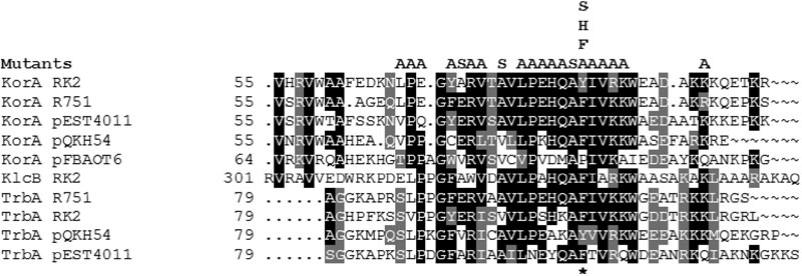
Alignment of conserved domains from KorA, KlcB and TrbA proteins. For KorA and TrbA proteins, only one representative sequence from each of the extant IncP-1 subgroups is included to improve clarity, along with the homologue of KorA from the IncU plasmid pFBAOT6 that is not thought to interact cooperatively with its cognate KorB. RK2, IncP-1; R751, IncP-1β; pEST4011, IncP-1δ; pQKH54, IncP-1γ; pFBAOT6 (IncU). The position of Tyr84 in RK2 KorA is indicated with an asterisk. Boxshade is used to indicate amino acids that are identical (black) and similar (grey) to the consensus. Amino acid substitutions created during the course of this study are indicated above the alignment.

The global transcriptional regulators of plasmid RK2 (also known as RP4), including repressors KorA, KorB, KorC and TrbA, form a complex network that coordinates expression of genes for all basic plasmid functions – replication, stable inheritance and transfer (Bingle and Thomas, 2001). KorB can engage in pairwise cooperative interactions with KorA or TrbA, resulting in enhanced repression of transcription when KorB and KorA or KorB and TrbA regulate a promoter together (Kostelidou *et al*., 1999; Zatyka *et al*., 2001; Bingle *et al*., 2003). KorA and KorB have been shown to cooperatively regulate the promoters for *korA* (autoregulation) and the replication gene *trfA* (Kostelidou *et al*., 1999) as well as putative stable inheritance gene *kfrA* (Bingle *et al*., 2005; Adamczyk *et al*., 2006). The synergy between KorB and KorA is also seen in cooperative binding of DNA by this pair of proteins (Kostelidou *et al*., 1999). The N-terminus of KorA contains a putative helix–turn–helix domain and has predicted structural similarity to many other bacterial transcriptional regulators (Kostelidou *et al*., 1998). KorA shares a conserved C-terminal domain (CTD) with TrbA repressor (Fig. 1) and this domain is also present in the middle of protein KlcB (function unknown) (Jagura-Burdzy *et al*., 1992; Larsen and Figurski, 1994). The CTD of TrbA and KorA is essential for cooperativity with KorB (Kostelidou *et al*., 1999; Zatyka *et al*., 2001) and it also has a role in dimerization (Bhattacharyya and Figurski, 2001). The mechanism that mediates the observed cooperativity has not been determined and is intriguing, given the flexibility observed in KorA:KorB and TrbA:KorB interaction, which appears to be unaffected by rotation of the operators relative to each other around the DNA axis or by the distance between the operators (Bingle *et al*., 2005). We have therefore used KorA as a model for understanding the interface with KorB as this protein is better characterized *in vitro* than TrbA and has also been the subject of structural studies (Rajasekar *et al*., 2006). In this paper we identify a key part of the interaction between KorA and KorB proteins by NMR coupled with mutagenesis of KorA followed by characterization of the mutant proteins.

## Results

### NMR shows that the CTD of KorA interacts specifically with KorB

**Fig. 2:**
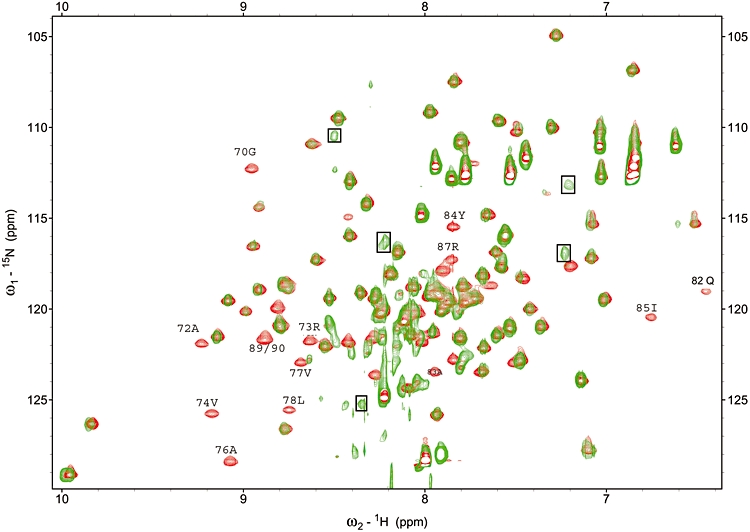
2D NMR analysis of KorA–KorB interaction. Overlay of ^1^H-^15^N HSQC of a solution containing a 2:1 ratio of ^15^N-KorA to unlabelled KorB (green) on free ^15^N-KorA (red). The free KorA residues that were shifted to a new position in the titration are marked with their residue number: 65, 70, 72, 73, 74, 76, 77, 78, 82, 84, 85, 87, 89 and 90. New peaks that were clearly due to amino acids in new environments are indicated with boxes, but have not been unambiguously assigned.

If there is a specific interaction between KorA and KorB, we expected that the interaction would occur in concentrated protein solution even in the absence of DNA. As we have completed the NMR backbone assignments for KorA (Rajasekar *et al*., 2006), it was possible to analyse the ^1^H-^15^N HSQC (Heteronuclear Single Quantum Coherence) spectrum of ^15^N-labelled KorA as unlabelled KorB was added gradually (Fig. 2). To simplify purification of KorB in NMR quantities, His-tagged KorB was used. This shows similar DNA-binding specificity in EMSA (electrophoretic mobility shift assay) and DNAse I footprinting (Lukaszewicz *et al*., 2002; Lukaszewicz, 2004) to wild-type (WT) KorB, and similar secondary structure as detected by CD spectrum and Analytical UltraCentrifugation – AUC (S.T. Muntaha, unpublished). In the HSQC spectrum peaks are seen for protons attached to ^15^N atoms from the main chain of the KorA protein or from side-chain amide and indole groups. KorB is not labelled with ^15^N and so no signals from KorB are observed. All the peaks broadened as KorB was added, indicating the formation of a complex that has a higher molecular mass than free KorA. Most of the peaks were otherwise unaffected by the presence of KorB, showing that their chemical environment is unchanged. However, 14 peaks seen in the free protein, all corresponding to amino acids concentrated in the C-terminal region, became less intense as KorB was added (65K, 70G, 72A, 73R, 74V, 76A, 77V, 78L, 82Q, 84Y, 85I, 87R, 89W and 90E) and, at the same time, new signals were observed that increased in intensity as KorB concentration increased (Fig. 2). The new peaks are from NH groups of KorA that are in a different chemical environment in the KorA–KorB complex from the corresponding groups in the free KorA. That two distinct peaks were observed for each amino acid, rather than a shift of the original peak to a new position, indicates that the two signals are in slow exchange. From the relative intensities of the ‘free’ and ‘bound’ peaks at increasing KorB concentration we estimated that each KorB dimer is able to contact two KorA dimers and at a molar ratio of 2 KorA: 1 KorB the signals from free KorA were no longer seen (Fig. 2). As KorA is dimeric, each peak in the KorA spectrum should correspond to two identical NH groups from the same amino acid in each subunit of the dimer. However, there was no evidence for the members of such a pair of residues in a KorA dimer behaving differently from each other. In fact, the contacts created at a ratio of 2 KorA: 1 KorB were sufficient to change the environment of all the affected KorA residues. This suggests that each KorB dimer can contact two KorA dimers – most simply one KorA dimer would be on each side of a KorB dimer – and that each of these KorB contact faces is sufficient to affect both monomers in a KorA dimer (Fig. 6). The results indicate that there are specific interactions between the proteins and that as predicted these are focused on the CTD of KorA.

### Tyrosine 84 is critical for cooperativity with KorB

In parallel with the NMR experiments, we mutated amino acids of the CTD to try to identify residues required specifically for cooperativity but not other functions of KorA. We screened a total of 23 residues in the CTD of KorA mainly by conversion to alanine, except for A72 and A76 that were converted to serine. The selection of residues was based on the degree of conservation of residues across sequenced KorA and TrbA homologues as well as those implicated in the interaction by NMR titration (Figs 1 and 2). Site-directed mutagenesis was carried out using mismatched polymerase chain reaction (PCR) primers in an *in vitro* overlap method as described in *Experimental procedures*. All mutant clones used were sequenced to check for PCR errors and the presence of the desired substitutions. Mutant proteins were expressed from the *tac* promoter (*tacp*) and screened for stability by Western blotting with KorA polyclonal antibodies. The detectable level of KorA E69A was less than 0.25× the level of WT KorA, while those for P79A and V86A were less than 0.1× the level of WT KorA. While this may be due to reduced antigenicity of the mutant proteins, the use of polyclonal antibodies makes this unlikely, so it is most likely that the reduced signals represent reduced levels of these three proteins, possibly due to an increased rate of degradation. All other proteins were produced at levels similar to the WT.

**Fig. 3:**
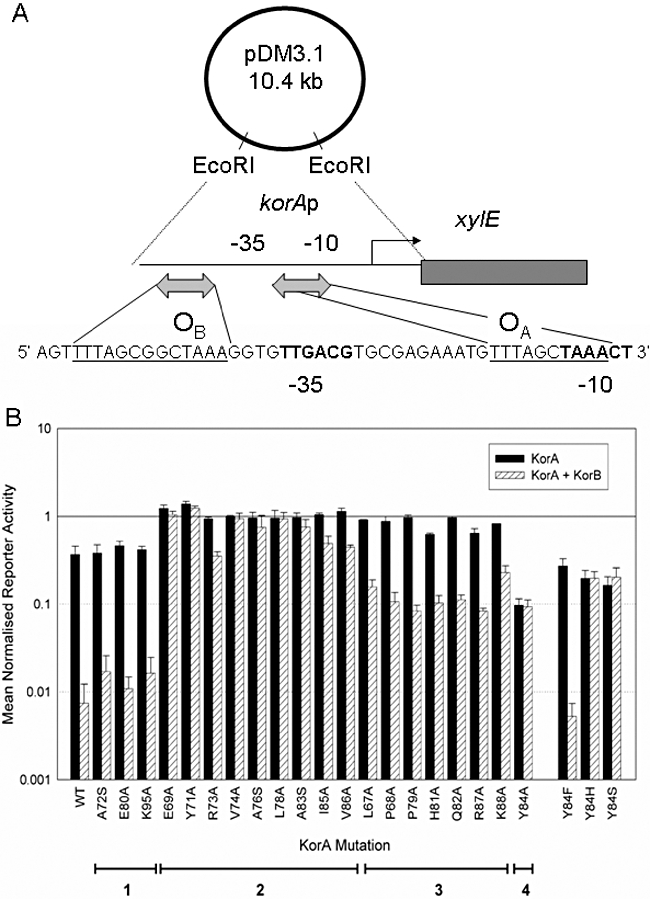
A. Scheme of the reporter plasmid pDM3.1 containing the *korA* promoter linked to the *xylE* reporter plasmid (Macartney *et al*., 1997). The sequence below shows the promoter region with KorB and KorA operators underlined (O_B_ and O_A_ respectively) as well as the the −35 and −10 boxes in bold. B. Phenotypes of KorA C-terminal domain mutants in reporter gene assays alone and in the presence of KorB. Bars indicate reporter gene activity from *korA* promoter relative to activity in the absence of any repressor. Black bars, in the presence of KorA; white bars, in the presence of KorA and KorB. Error bars indicate standard deviation. Mutants are grouped by similar phenotype.

Reporter gene (*xylE*) assays with WT and mutant KorA proteins in the absence and presence of KorB were used to screen for mutations that might specifically affect the cooperativity interface. The reporter gene construct used (pDM3.1, Fig. 3A) carries the *korA* promoter (*korAp*) firing into the *xylE* reporter gene. At *korAp*, a KorA operator (O_A_– TTTAGCTAAA) overlaps the −10 region of the promoter and a KorB operator (O_B_– TTTAGC^c^/_g_GCTAAA) lies just upstream of the −35 region (32 or 33 bp centre–centre distance depending on whether one counts the central nucleotide of O_B_ as 0 or 1). KorB and KorA proteins were supplied *in trans* from *tacp* expression vectors. The assay cultures were grown without IPTG induction as we have previously established that this gives a more ‘natural’ low level of KorB in the cell (Bignell *et al*., 1999) and does not result in any significant detectable level of repression by KorB alone (mean activity in the presence of KorB alone was 1.07× activity in the absence of KorB). The levels of activity were normalized relative to activity of *korAp* in the absence of either KorA or KorB. As in previous experiments the activity of the reporter gene is repressed only weakly in the presence of KorA alone (about threefold at this level of induction) but in the presence of both KorA and KorB the reduction is more than 100-fold (Bingle *et al*., 2005) although it should be noted that in this previous study we used a low level of induction with 0.05 mM IPTG. From these results (Fig. 3B) the *korA* mutants could be divided into four groups. Group 1 mutants (A72S, E80A and K95A) are essentially wild-type. Group 2 mutants (E69A, Y71A, R73A, V74A, A76S, L78A, A83S, I85A and V86A) are defective repressors showing less than twofold repression when acting alone and less than threefold repression in the presence of KorB. As discussed above Western blotting of uninduced expression cultures was carried out on all mutants and this indicated that three KorA mutant proteins in group 2 were produced at low levels: E69A, P79A and V86A. All other group 2 mutant proteins were expressed at levels similar to WT, suggesting that the mutated residue affects multiple properties, either because of direct involvement or due to structural changes in the protein that have knock-on effects. As L78 was identified by Bhattacharyya and Figurski (2001) as essential for dimerization, it is possible that the phenotype of this and the other group 2 mutants arises from irreversible loss of dimerization. Group 3 mutants (L67A, P68A, P79A, H81A, Q82A, R87A, K88A) do not repress alone (less than twofold repression) but are capable of threefold or greater repression together with KorB. These mutations may affect dimerization but allow partial suppression of the defect by the interaction with KorB. Group 4 is represented by a single mutant (Y84A) that has a WT (or better) phenotype for repression when acting alone, but is not potentiated by KorB, suggesting that this tyrosine residue is critical for the normal KorA–KorB interaction. As this was the most interesting of the mutant types it was the primary focus of further work.

### Tyrosine 84 can be replaced by phenylalanine without loss of function

Comparison of the sequences of KorA and TrbA proteins (Fig. 1) showed that all of the IncP-1 proteins have either tyrosine (Y) or phenylalanine (F) at the position equivalent to Y84 in RK2 KorA. These residues appear to be interchangeable as there is no consistent pattern by protein (KorA or TrbA), or plasmid, that might suggest significant changes to the KorA–KorB interface. We therefore introduced into RK2 KorA three further changes – phenylalanine (F), serine (S) and histidine (H) at this position, the logic being to create the known variant (F) as well as amino acids with either an hydroxyl group but not an aromatic ring (S), or a five membered aromatic ring (H). The results showed that F was as good as Y in the repression assays, but that H or S resulted once again in loss of cooperativity without loss of repression (Fig. 3B).

### Co-purification confirms direct interaction between KorA and KorB and importance of KorA residue Y84

**Fig. 4:**
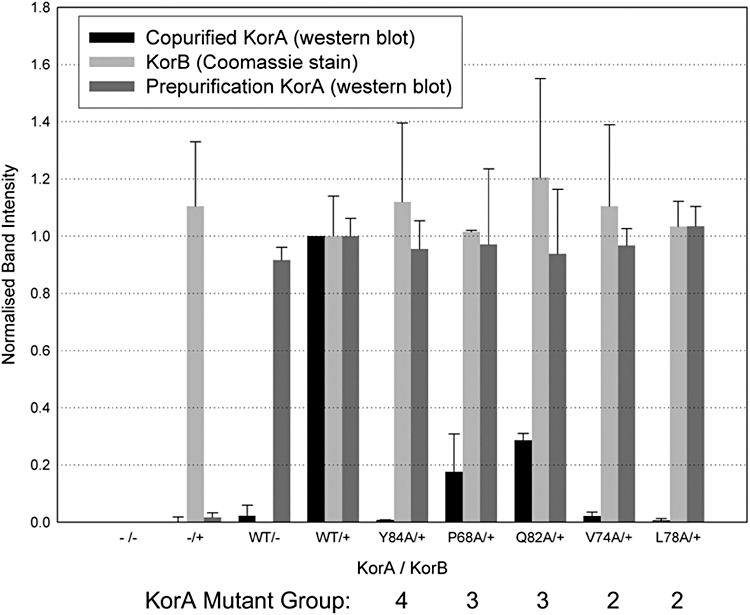
Co-purification of KorA with His-tagged KorB. Cell extracts with WT or mutant KorA and His-KorB alone were mixed, then Ni-NTA added and His-KorB purified. Samples of the extracts or the purified His-KorB were fractionated by SDS-PAGE, stained with Coomassie blue to detect His-KorB, Western-blotted and probed with purified anti-KorA antiserum. Labels on the horizontal axis indicate presence or absence of KorA/His-KorB in lysates. Negative controls were: extracts lacking KorA (−/− and −/+), which showed no KorA signal in either the presence or the absence of His-KorB; and extracts with KorA mixed with Ni-NTA in the absence of His-KorB (WT/–), which showed negligible retention of KorA. Band intensity values were normalized to those obtained with WT KorA/His-KorB.

As the NMR data indicate that KorA and KorB undergo direct contact, we performed pull-down assays to determine whether the interactions were strong enough to result in co-purification of the two proteins. Crude lysate of bacteria expressing His-tagged KorB was mixed with crude lysate from cells expressing WT or mutant KorA. Nickel agarose (NiNTA) was added and His-tagged KorB protein purified by spinning out the gel and washing to remove non-specifically bound proteins. Western blotting was used to detect KorA in the original extract and co-purified with His-KorB. The experiment was performed this way round because even non-His-tagged KorB can be retained on Ni-Agarose whereas KorA does not bind unless His-tagged. WT KorA was found to co-purify on Ni-NTA with His-tagged KorB, thereby confirming a direct protein–protein interaction (Fig. 4), whereas Y84A showed little or no co-purification with KorB. When other selected mutant proteins were compared, class 2 mutants (for example V74A and L78A that are completely defective as repressors even in the presence of KorB) did not co-purify with KorB, whereas class 3 mutants (for example P68A, Q82A and P79A that are defective as repressors alone but exhibit some activity in the presence of KorB) showed reduced co-purification with KorB (Fig. 4). These results confirm that Y84 is critical for the correct formation of the interaction interface.

### KorA Y84A still binds DNA *in vitro* but is defective in cooperativity with KorB

**Fig. 5:**
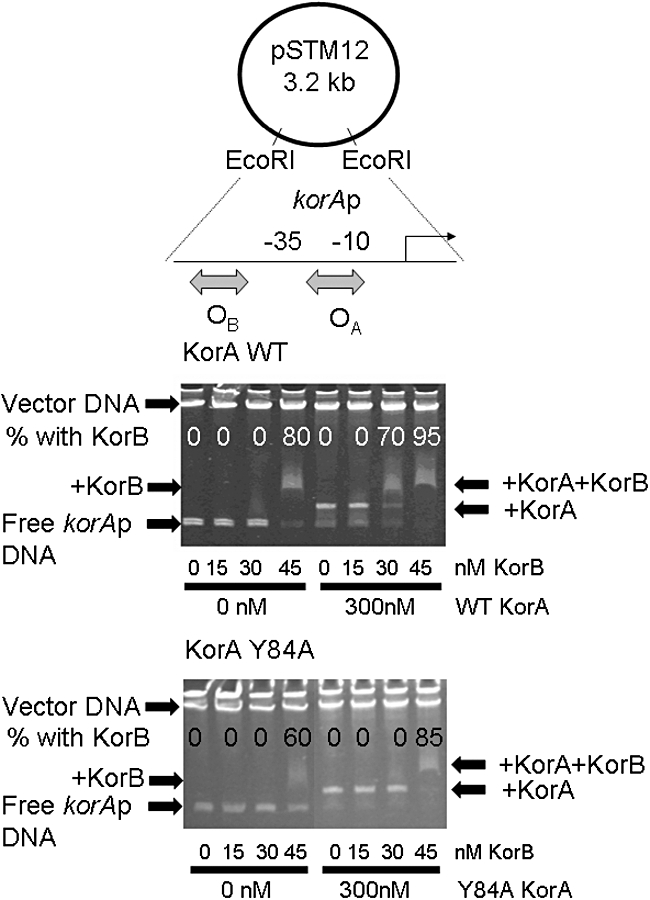
KorB electrophoretic mobility shift of a 220 bp *korA*p DNA fragment, in the absence and presence of KorA WT and Y84A. The *korAp* fragment was released from pSTM12 by EcoRI digestion – the 2.6 kb vector fragment runs at the top of the gel. The *korA* promoter includes KorA (O_A_: 5′-GTTTAGCTAAAC-3′) and KorB (O_B_: TTTAGCCGCTAAA-3′) sites separated by 20 bp. Each protein had previously been titrated separately to determine suitable concentrations. Proteins were added separately or together as described in *Experimental procedures*. The percentage of DNA fragment in the KorB–DNA complex, with or without KorA, is indicated on the gel. At 30 nM KorB the presence of 300 nM WT KorA results in > 80% retardation of the KorAp–KorA complex whereas the presence of 300 nM Y84A KorA has no effect.

We have previously demonstrated that *in vitro* KorB binds preferentially to DNA with KorA bound rather than to free operator (Kostelidou *et al*., 1999). To check whether the Y84A mutation affects this interaction with KorB in an electrophoretic mobility shift assay, WT and Y84A mutant His-tagged KorA as well as KorB were purified as described in *Experimental procedures*. They were tested using EcoRI-cut plasmid pSTM12 DNA that releases an approximately 220 bp *korA*p fragment with KorA and KorB operators separated by a centre–centre spacing of 32/33 bp. A key difference from our previous assays is that we used non-radioactively labelled DNA in this work, visualizing the DNA by ethidium bromide fluorescence, so the DNA concentrations used were higher and thus the amount of protein needed for retardation appears to be higher than we have reported previously (Kostelidou *et al*., 1999). Also the fragment was not purified so the vector fragment was also present. Initially, the amounts of both KorA and KorB were varied separately to identify the concentration at which KorA gave partial retardation and the concentration at which KorB first gave significant retardation. KorB binding alone had a *K*_app_ of about 40–45 nM (Fig. 5). In the absence of KorB, KorA Y84A consistently showed lower *K*_app_ of about 200 nM compared with 300 nM for WT KorA, in agreement with the enhanced repression by the mutant KorA observed in reporter assays (Fig. 3), but for both proteins 300 nM still gave a mixture of unbound and bound DNA fragments (Fig. 5). When WT KorA was present, KorB binding to the *korA*p DNA fragment was reproducibly enhanced, *K*_app_ being reduced to 20–25 nM. In addition, the intensity of the DNA plus WT KorA band was decreased more strongly than the free DNA band, indicating an approximately threefold preference for the DNA–KorA complex, which is slightly higher than the approximately twofold preference previously reported (Kostelidou *et al*., 1999). By contrast, the presence of KorA Y84A had consistently less effect on KorB binding (Fig. 5), indicating that the enhancement in KorB binding to DNA by WT KorA has been weakened by the Y84A mutation.

## Discussion

The results presented in this paper demonstrate that there is a direct and specific interaction between KorB and amino acids in the C-terminal region of its co-regulator KorA. This interaction could be detected in purified solutions of KorA and KorB (Fig. 2), as well as cell extracts (Fig. 4). This is in line with our previous predictions based on the conservation of this region between the two repressor proteins, KorA and TrbA, which show cooperativity with KorB and with deletion studies on these proteins that implicated the CTD as important for this (Kostelidou *et al*., 1999; Zatyka *et al*., 2001). Our mutational analysis of the region of KorA involved in this interaction identified one residue, tyrosine 84, as not essential for repressor activity of KorA (Fig. 3), but essential for the interaction between KorA and KorB and allowed us to connect the *in vitro* interaction between KorA and KorB (seen by pull-down and EMSA) with the ability of KorA and KorB to cooperate in repression. Other amino acids may be involved in the interaction between the two proteins as indicated by the NMR titration, but other mutations that affected cooperativity also affected the repressor function of KorA, so conclusions can not be drawn about how important they are for the protein–protein interaction. Other amino acids being involved would be consistent with our previous work on KorA in which one of the two deletion derivatives created, which had lost aa 84–101, still retained some cooperativity in DNA binding with KorB *in vitro* (Kostelidou *et al*., 1999). However, as deletion derivatives, especially those in a dimerization domain (Bhattacharyya and Figurski, 2001), are likely to alter gross structure, their properties may not reflect the normal interactions of the full-length protein. It is noteworthy that 8 of the 9 residues identified by *in vivo* analysis as being important for cooperativity were shown to shift in the NMR analysis of KorA–KorB interaction (the exception being Y71), whereas only 2 out of 7 residues identified by *in vivo* analysis as being non-essential for cooperativity were shifted in the NMR analysis. Consistent with all these observations is the fact that the ability of KorA to potentiate KorB binding to a DNA fragment containing *korA*p with suitably spaced operators for KorA and KorB was reduced by the Y84A mutation (Fig. 5). Given the dual role that this region of KorA appears to play (i.e. dimerization and cooperativity), we may be fortunate in finding a residue that only appears to affect one of the two major functions and that seems to have such a marked effect on cooperativity as a degree of individual redundancy has been observed in the amino acids involved in other protein–protein interfaces (Jobichen *et al*., 2007).

**Fig. 6:**
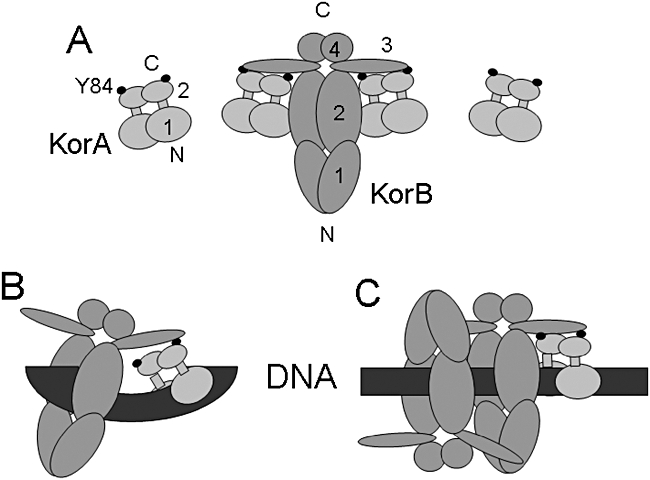
Scheme to summarize possible KorA–KorB interactions. For KorA the DNA binding (aa 1–61) and dimerization (aa 68–101) domains are shown as 1 and 2, respectively, while for KorB the apparently flexible N-terminal domain (aa 1–138), the DNA binding domain (aa 139–252), a putative flexible linker (aa 253–298) and the C-terminal dimerization (aa 299–358) domains are labelled 1, 2, 3 and 4 respectively. A. In solution, KorB can titrate the KorA NMR signals at a ratio of 1 KorB: 2 KorA so the complex is shown with this stoichiometry. However, on DNA the presence of specific binding sites constrains the location of adjacent KorA and KorB dimers and contact may be made by looping (B) or spreading (C) in a flexible way as rotation of the binding sites through 180° to each other does not interfere with observed cooperativity (Bingle *et al*., 2005).

That tyrosine 84 can be changed to phenylalanine without loss of cooperativity, while changes to serine or alanine inactivated this function, rules out modification of the tyrosine (for example by phosphorylation) as important for the interaction, as this normally depends on the hydroxyl group. Thus by elimination it is likely to be the benzene ring that is critical for the interaction with KorB. We therefore predict that Y84 protrudes from KorA so that it can contact a suitable part of the surface of KorB. From the titration with increasing KorB concentration it appears that each KorB dimer can contact two KorA dimers and that this contact affects the signal for both Y84 residues in each KorA dimer (Fig. 6A). This is consistent with KorB having two sides, each of which can simultaneously contact a KorA dimer, possibly contacting both subunits at once or singly but in fast exchange. In the presence of DNA, the KorA and KorB dimers will be oriented by their operators, and we assume only a 1:1 complex is formed. The predicted size of KorA (101 aa; monomer diameter if approximately spherical of 2.5 nm), the known size of the KorB DNA binding domain (aa 140–250 as a monomeric, slightly extended sphere with a diameter of approximately 3 nm; Khare *et al*., 2004) and the DNase I foot-printing of KorA and KorB (Williams *et al*., 1993; Jagura-Burdzy and Thomas., 1995) on *korA*p when their operators are present with 33 bp between their centres are all consistent with them being separated by approximately two turns of the DNA helix (about 7 nm). Dimers are therefore likely to be close but not in direct surface-surface contact unless one or both proteins have an extended rather than a spherical structure. As neither repression nor cooperativity is affected by insertion of an additional 5 bp between the operators that should move the relative positions of these proteins by 180° (Bingle *et al*., 2005), it seems unlikely that the proteins are relatively rigid and interact simply by KorA- or KorB-induced DNA bending (Fig. 6B). This implies that the patch on KorB with which KorA interacts is either present in multiple copies, on different faces of the KorB surface, or is in a sufficiently flexible position (e.g. on a flexible linker) to allow multiple architectures for the interaction. Alternatively, it may be that the KorB spreads from its operator by recruiting additional KorB dimers in such a way that they provide for flexible interaction (Fig. 6C). As KorA can potentiate silencing by KorB even when its binding site lies between the KorB binding site and the target for silencing (Chiu *et al*., 2008), it seems likely that the higher order complexes that KorB can make with DNA and KorA are complex and therefore simple models for a tertiary protein–protein–DNA complex may not be applicable.

Tyrosine is often found to be involved in protein–protein interactions, either through the aromatic ring or the hydroxyl group. In transcriptional regulation a tyrosine residue in the C-terminal domain of the α-subunit (α-CTD) of RNA polymerase is critical for regulatory interaction with Spx, a global transcription regulator from *Bacillus subtilis* (Newberry *et al*., 2005), while Y8 in the elongation factor RfaH is critical for its interaction with the β′-subunit of RNA polymerase (Sevostyanova *et al*., 2008). A dependence on multiple conserved tyrosine residues has also been observed in transcriptional activation by the EWS/ATF1 oncogene (Feng and Lee, 2001). In a different context, Y548 in the P16 domain of *Bacillus stearothermophilus* DnaG primase is critical for the interaction with DnaB helicase (Bailey *et al*., 2007; Chintakayala *et al*., 2008), but in this example it appears that the phenolic hydroxyl group is a critical part of the interaction, contacting the amide oxygen of N101 in the N-terminal region of DnaB. How the aromatic ring of Y84 interacts with KorB will therefore be of considerable interest.

It may be significant that in the closest KorA relatives encoded by plasmids outside the IncP-1 plasmid family, KorA of the *Sinorhizobium meliloti* plasmid pMBA19a (Watson and Heys, 2006) and TrbB (another name for KorA) of IncU family plasmid pFBAOT6 (Rhodes *et al*., 2004), the amino acid at the equivalent of this position is neither Y nor F, while within the IncP-1 family it is always one or other of these two amino acids (Fig. 1). That this residue is not conserved in the CTD of KorA in IncU plasmids is consistent with the lack of a cooperative interaction between the KorB and KorA homologues of IncU plasmid RA3, which is essentially identical to pFBAOT6 (A. Kulinska and G. Jagura-Burdzy, pers. comm.). As plasmids of other groups also possess paralogues of both KorA and KorB (for example Greated *et al*., 2000; 2002; Schneiker *et al*., 2001) and lack this residue in their KorA homologue, it seems plausible that the interaction between KorA and KorB evolved in IncP-1 plasmids. This might have followed accumulation and shuffling of multiple regulator binding sites, as deduced recently for a set of related eukaryotic genome sequences, which revealed how flexible the positions of regulator binding sites can be over evolutionary time, producing different combinations of multifactorial regulation in different species (Tuch *et al*., 2008). However, within the IncP-1 plasmids, the strong similarity of the *trfA*, *korA* and *klcA* promoter regions and their similarly spaced KorA and KorB operators (Smith *et al*., 1984; Pansegrau *et al*., 1994) suggests that they may have been propagated as preformed units, as is clear must have happened for the *klcA*, *kleA* and *kleC* promoter regions (Thomas *et al*., 1988). The dimerization and KorB cooperativity domain could thus have evolved on KorA in the context of closely spaced KorB and KorA binding sites, and then this domain could have been acquired by the DNA binding domain of TrbA, allowing it to interact with KorB at binding sites located at much more variable distances (Bingle *et al*., 2003; 2005). As the majority of IncP-1 KorA/TrbA proteins have Phe rather than Tyr at this position, it may be that Phe was the original amino acid, although the codons for these amino acids (*F* = UU^U^/_C_ and Y = UA^U^/_C_) are easily interconverted by changing their central nucleotide from U to A. The fact that the change to Phe or Tyr at this position may have made KorA a weaker repressor when acting alone was presumably compensated for by the stronger repression achievable by the cooperativity of KorA and KorB. This, however, would also mean that loss of either repressor would subsequently be strongly disfavoured. Thus appearance of cooperativity would be a further step in the development of integration and co-dependancy of regulatory circuits exhibited by the evolution of the IncP-1 family – tight repression allows very strong promoters to exist but makes survival increasingly dependent on these repressors. Experiments currently underway are exploring the consequences for the plasmid of destroying this cooperative interaction to determine just how advantageous it is for the survival and competitiveness of the plasmid.

## Experimental procedures

### Bacterial strains and growth conditions

The *E. coli* K12 strain used for DNA manipulations was DH5α[F^-^*supE44*Δ*lac*U169 (φ80 lacZ ΔM15) *hsdR*17 *recA*1 *endA*1 *gyrA*96 *thi-*1 *relA*1] (Grant *et al*., 1990). Proteins were expressed in *E. coli* BL21(DE3) [F^-^*ompT gal* (*dcm*) (*met*) (*lon*) *hsdS*_*B*_ (DE3)] (Studier *et al*., 1990). XylE assays were carried out in *E. coli* K12 strain C600K^-^[F^-^*thr-1 leu-6 thi-1 lacY1 supE44 tonA21 galK3*] (McKenney *et al*., 1981). Bacteria were grown in L-broth (Kahn *et al*., 1979) at 37°C with shaking at 200 r.p.m.; solid media were obtained by the addition of agar at 1.5%. Antibiotics were added, when required, at the following concentrations: ampicillin 100 μg ml^−1^; kanamycin 50 μg ml^−1^, streptomycin 30 μg ml^−1^.

### DNA purification, cloning and sequencing

Plasmid DNA was prepared by an alkaline-lysis miniprep method (Sambrook and Russell, 2001) or, when a higher level of purity was required, using the Wizard Plus SV miniprep kit (Promega) or Plasmid Midi kit (Qiagen). Enzymic manipulations of DNA were carried out using standard techniques (Sambrook and Russell, 2001) or according to the manufacturer's instructions. DNA sequencing was performed on an automated 373A DNA Sequencer or 3700 DNA Analyser (Applied Biosystems) using a dye-terminator kit (Big-Dye; PE Applied Biosystems).

### PCR and mutagenesis of *korA*

Polymerase chain reaction was performed using the proof-reading DNA polymerase KOD (Novagen). Thermal cycling was performed as recommended by the manufacturer. *In vitro* overlap PCR (Ling and Robinson, 1997) to introduce internal mutations into a DNA fragment was performed as follows. Overlapping, mismatched, internal primers (listed in Table S1) were used in separate reactions with corresponding external primers on a WT template. The products from this reaction were purified from an agarose gel (to remove the original template) using the GeneClean kit (Bio101) and then combined, denatured, annealed and extended *in vitro* during two rounds of thermal cycling. These extension products, incorporating the desired mutations, were amplified in a second PCR reaction using only the external primer pair and then cloned in T-tailed vector pGEM-T Easy (Promega). After the sequence was checked the mutated gene was recloned into appropriate expression plasmids – either pGBT30 (Jagura-Burdzy *et al*., 1991) for *xylE* reporter gene assays or pET28A derivative pGBT340 (Jagura-Burdzy *et al*., 1999) lacking the T7 tag for overexpression and purification.

### Protein expression and purification

For NMR the *korA* gene was cloned between the NcoI and HindIII sites down stream of the T7 promoter of a pET28a (Invitrogen) derivative pGBT340 (Jagura-Burdzy *et al*., 1999) to give pLB86 (Rajasekar *et al*., 2006) and expressed in *E. coli* BL21 (λDE3). Bacteria were grown to an OD_600_ = 0.6 in 1 l M9 minimal medium with ^15^NH_4_Cl and ^13^C_6_H_12_O_6_ as the sole nitrogen and carbon sources, respectively, induced with 1 mM IPTG and then harvested in stationary phase by centrifugation. The pellet was suspended in 20 mM Tris HCl at pH 7.5 at RT, 1 mM EDTA, 1 M NaCl, 10 μg ml^−1^ DNAseI, 10 μg ml^−1^ RNAse, 10 μg ml^−1^ PMSF (Phenyl methyl sulfonyl fluoride, a serine protease inhibitor) and protease inhibitor cocktail (215 mg/20 g cells, Sigma P8465). Bacteria were lysed by sonication for 8 × 15 s at a frequency of 20 kHz and broken cells pelleted by centrifugation at 32000 *g* for 30 min. After clearing proteins precipitated at 30% ammonium sulphate KorA was enriched by precipitation at 75%, re-suspended in 100 mM Tris HCl pH 7.0, 5 mM EDTA and 10 μg ml^−1^ PMSF and dialysed against 20 mM sodium phosphate at pH 7.6, 100 mM NaCl, 1 mM EDTA and 10 μg ml^−1^ PMSF. The dialysed extract was loaded on to a Hi Trap^TM^ SP Sepharose 5 ml column (a strong cation exchange resin equilibrated in 20 mM sodium phosphate buffer at pH 7.6 containing 100 mM NaCl) and eluted by NaCl gradient (100–600 mM). The eluted KorA was loaded on to a Heparin agarose HiTrap^TM^ 5 ml column (17-0407-01 GE lifesciences) equilibrated in 20 mM sodium phosphate buffer at pH 7.6, 100 mM NaCl and 1 mM EDTA. The pure fractions eluted from the Heparin column were pooled and concentrated by ultrafiltration. Protein quantification was carried out by absorbance at 280 nm using calculated molar absorbance (Pace *et al*., 1995).

For electrophoretic gel mobility shift experiments His_6_-KorA-WT, His_6_-KorA-Y84A and His_6_-KorB were overexpressed in C600 BL21 from a modified pET28a vector pGBT340 (Jagura-Burdzy *et al*., 1999). Cultures of 400 ml were grown to mid-log phase (OD_600nm_∼0.4–0.6) at 37°C and induced with 1 mM IPTG for 4 h, prior to harvesting the cells at 18 600 *g*. Purification was carried out using a Ni-NTA column (QIAGEN), according to the manufacturer's instructions for purification under native conditions and then the protein solutions were dialysed to remove imidazole. The His_6_-KorB was also used in the NMR experiments.

### NMR titration experiments

^15^N-KorA and His_6_-KorB were dialysed against 10 mM sodium phosphate buffer pH 7.0, 0.1 mM EDTA, 100 mM NaCl and mixed in the appropriate ratios. The 2D ^1^H-^15^N HSQCs were carried out at 25°C in 500 MHz Bruker Spectrometer with cryoprobe. Exactly 1024 and up to 128 points were collected in the direct (t1) and indirect (t2) dimensions respectively. The 2D experiments were processed using an adjustable sine window in both dimensions and zero filling to 2048 and up to 128 complex points in t1 and t2 dimension respectively. Processing was carried out using NMRPIPE (Delaglio *et al*., 1995) software. Peak picking and analysis was carried out by CCPN (Vranken *et al*., 2005).

## XylE reporter gene assays

The level of expression of the *xylE* reporter gene was determined by an enzymatic assay of activity of the gene product (catechol 2,3-oxygenase) in logarithmically growing bacterial cultures (Zukowski *et al*., 1983). One unit is defined as the amount of enzyme necessary to convert 1 mmol of substrate to product in 1 min under standard conditions. Protein concentration was determined by the Biuret method (Gornal *et al*., 1949). KorA repressor protein was expressed from pMB1-based *tacp* expression constructs based on pGBT30 carrying WT *korA* (Jagura-Burdzy *et al.*, 1991) or mutant *korA*s (this study) and KorB from the IncQ *tacp* expression vector pDM1.21 (Macartney *et al*., 1997). The corresponding empty vector controls were pGBT30 (Jagura-Burdzy *et al*., 1991) or pDM1.2 (Macartney *et al*., 1997). Repression index ratios were calculated as XylE activity in absence of repressor/XylE activity in presence of repressor. Cooperativity index was calculated as the repression index obtained with two repressors present divided by the product of the repression indices for single repressors. Standard deviations were estimated for repression indices by error propagation from the standard deviations of XylE activity measurements. Means and standard deviations were calculated from two or three replicates. Data shown are typical of at least two independent experiments.

## Co-purification/pull-down assay for protein–protein interaction

Wild-type and mutant KorA proteins were expressed from pMB1 *tacp* expression plasmids as described above (pGBT30 negative control). KorB was expressed with an N-terminal His_6_-tag from a T7 promoter on the pET28a derivative pSMB320 (Batt, 2008). The two interaction partners were expressed separately and then expression cultures combined prior to lysis and purification. Overnight cultures were grown in L-broth supplemented with appropriate selective antibiotic(s) and 1% glucose to minimize leaky expression from the T7 promoter of pET-based constructs. Overnight cultures were diluted 1/100 and grown on at 37°C, 200 r.p.m. until OD_600_ = 0.6–1.0, before induction with 0.5 mM IPTG. Proteins were expressed for about 4 h before combining 1 ml samples in appropriate pairwise combinations and harvesting cells by centrifugation for 1 min at 15 000 *g* (at room temperature). Cell pellets were re-suspended in 200 μl lysis buffer (50 mM NaH_2_PO_4_, 300 mM NaCl, 10 mM imidazole, pH 8), lysozyme was added to 1 mg ml^−1^ and the suspension was incubated on ice for 30 min. Cells were lysed by brief (20 s) sonication and the lysate was cleared by centrifugation for 10 min at 15 000 *g* (at 4°C). Twenty microlitres of a 50% slurry of Ni-NTA resin (Qiagen) was added to each tube, and mixed gently on a rotator for 30 min at 4°C. Resin and bound proteins were pelleted by centrifugation for 20 s at 5000 *g* and then washed twice with 100 μl wash buffer (50 mM NaH_2_PO_4_, 300 mM NaCl, 20 mM imidazole, pH 8). Finally, the bound proteins were eluted with 60 μl of elution buffer (50 mM NaH_2_PO_4_, 300 mM NaCl, 250 mM imidazole, pH 8). Elution and pre-purification samples were separated on 15% SDS-PAGE and transferred to nitrocellulose membrane (Hybond-C Extra, Amersham) using the Mini Trans-Blot Electrophoretic Transfer Cell (Bio-Rad). The KorA bands were detected by Western blotting using purified anti-KorA antiserum (supplied by Sarah Batt) and the Amplified Alkaline Phosphatase Goat Anti-rabbit Immuno-Blot kit with colorimetric (BCIP/NBT) detection (Bio-Rad). His-KorB was detected by staining duplicate gels with colloidal Coomassie blue stain (ProtoBlue Safe, National Diagnostics). Band intensities corresponding to His-KorB from Coomasssie-stained gels or KorA proteins from Western blots were quantified using a GS-710 Calibrated Imaging Densitometer and Quantity One software (Bio-Rad).

## Electrophoretic mobility shift assays (EMSA)

DNA binding reactions were set up in 20 μl buffer (50 mM NaCl, 10 mM Tris-HCl, 10 mM MgCl_2_, 1 mM Dithiothreitol, pH 7.9) with approximately 1 nM linear DNA fragments from EcoRI-cut plasmid pSTM12 (this paper), which consists of a PCR-amplified 220 bp *korA* promoter segment (primers used: forward, 5′-CCTCCTGGAACTGGCTTTCGG-3′ and reverse 5′-AGCCATAAGCGGCAAGAGACG-3′) cloned in pGEMTeasy (Promega). Specified proteins were added to the concentrations shown and incubated at 37°C for 15 min. Standard bromophenol blue/glycerol sample buffer was added and the samples were loaded on 5% (w/v) polyacrylamide gels in 1× TBE buffer (Sambrook and Russell, 2001) and run at 4°C. The gel was soaked in dilute ethidium bromide solution (1 μg ml^−1^) and the DNA visualized under UV light. Quantity One software (Bio-Rad) was used to determine the band intensity of each sample on the gel.
