# Supplementary material for: A single aromatic residue in transcriptional repressor protein KorA is critical for cooperativity with its co-regulator KorB
Source: Mol Microbiol. 2008 Oct 29;70(6):1502–14. doi: 10.1111/j.1365-2958.2008.06498.x (PMC2680271; doi:10.1111/j.1365-2958.2008.06498.x)
Supplement: Supplementary file 1 [file mmi0070-1502-SD1.pdf]

Supplementary Table S1. Primers used for PCR mutagenesis of *korA*.

Primer suffixes: F, forwards; R, reverse. In each PCR, flanking primers ECO_KORA_F and KORA_SAL_R where used in combination with a pair of overlapping F and R central (mutagenic) primers. All oligonucleotides are written in 5'-3' orientation. The mutagenised nucleotide is shown in lower case font. Not all changes have F and R primers, as sometimes the F primer for one mutation was used in combination with the R primer for a different mutation, the two products being segregated on cloning. To make mutations towards the 3' end of *korA* (e.g. K95A), a standard PCR reaction was carried used with ECO_KORA_F in combination with a long mutagenic R primer.

Name	Sequence
ECO_KORA_F	CGAATTCATGAAGAAACGGCTTACC
KORA_SAL_R	CGTCGACTCATCGTTTGGTTTCCTG
KORA_L67A_F	CCCTCGGGCgcGTTCTTGTCCTCG
KORA_P68A_F	GAGGACAAGAACTTGgCCGAGG
KORA_P68A_R	CCTCGGcCAAGTTCTTGTCCTCG
KORA_E69A_F	AACTTGCCCGcGGGGTACGCGC
KORA_Y71A_F	GCCCGAGGGGgcCGCGCGGGTAAC
KORA_Y71A_R	GCGCGgcCCCCTCGGGCAAGTTC
KORA_A72S_F	CGAGGGGTACtCGCGGGTAACG
KORA_R73A_F	AGGGGTACGCGgcGGTAACGGCGGTTC
KORA_V74A_F	TACGCGCGGGcAACGGCGGTTC
KORA_V74A_R	GGCAGAACCGCCGTTgCCCGCGCGTACCCCTCG
KORA_A76S_R	CCGGCAGAACCGaCGTTACCCGC
KORA_L78A_R	CCTGATGTTCCGGCgcAACCGCCGTTACCCG
KORA_P79A_F	GGTTCTGgcGGAACATCAGGCGTAC
KORA_E80A_F	CGGTTCTGCCGGcACATCAGGCG
KORA_E80A_R	CGCCTGATGTgCCGGCAGAAC
KORA_H81A_F	CTGCCGGAAgcTCAGGCGTACATCG
KORA_H81A_R	GTACGCCTGAgcTTCCGGCAGAACC
KORA_Q82A_F	CCGGAACATgcGGCGTACATCGTCCGG
KORA_A83S_F	GGAACATCAGtCGTACATCGTCCG
KORA_Y84A_R	TTCCGGACGATGgcCGCCTGATGTTCC
KORA_Y84H_F	ACATCAGGCGcACATCGTCCGGA
KORA_Y84F_R	CTTCCGGACGATGaACGCCTGATG
KORA_Y84S_R	CTTCCGGACGATGgACGCCTGATG
KORA_I85A_R	TCCCACTTCCGGACGgcGTACGCCTG
KORA_V86A_R	CTTCCGGgCGATGTACGCCTGATG
KORA_R87A_F	GGCGTACATCGTCgcGAAGTGGGAAG
KORA_R87A_R	TCCCACTTCgcGACGATGTACGCC
KORA_R87D_F	GGCGTACATCGTCgacAAGTGGGAAG

KORA_R87E_R	TTCCCACTTCtcGACGATGTACGCC
KORA_R87L_R	TCCCACTTCaGGACGATGTACGCC
KORA_K88A_F	GCGTACATCGTCCGGgcGTGGGAAGCG
KORA_K95A_R	GTCGACTCATCGTTTGGTTTCCTGTTTTgcCTTGGCGTC
